# Improved genomic prediction using machine learning with Variational Bayesian sparsity

**DOI:** 10.1186/s13007-023-01073-3

**Published:** 2023-09-02

**Authors:** Qingsen Yan, Mario Fruzangohar, Julian Taylor, Dong Gong, James Walter, Adam Norman, Javen Qinfeng Shi, Tristan Coram

**Affiliations:** 1 Australian Institute for Machine Learning, University of Adelaide, Adelaide, Australia; 2 School of Food, Agriculture and Wine, University of Adelaide, Adelaide, Australia; 3 School of Computer Science and Engineering, The University of New South Wales, Sydney, Australia; 4 Australian Grains Technologies, Roseworthy, Australia; 5 School of Computer Science, Northwestern Polytechnical University, Xi’an, China

**Keywords:** Machine learning, Genomic prediction, Linear mixed models, Bayesian, Variational inference, Feature selection

## Abstract

**Background:**

Genomic prediction has become a powerful modelling tool for assessing line performance in plant and livestock breeding programmes. Among the genomic prediction modelling approaches, linear based models have proven to provide accurate predictions even when the number of genetic markers exceeds the number of data samples. However, breeding programmes are now compiling data from large numbers of lines and test environments for analyses, rendering these approaches computationally prohibitive. Machine learning (ML) now offers a solution to this problem through the construction of fully connected deep learning architectures and high parallelisation of the predictive task. However, the fully connected nature of these architectures immediately generates an over-parameterisation of the network that needs addressing for efficient and accurate predictions.

**Results:**

In this research we explore the use of an ML architecture governed by variational Bayesian sparsity in its initial layers that we have called VBS-ML. The use of VBS-ML provides a mechanism for feature selection of important markers linked to the trait, immediately reducing the network over-parameterisation. Selected markers then propagate to the remaining fully connected feed-forward components of the ML network to form the final genomic prediction. We illustrated the approach with four large Australian wheat breeding data sets that range from 2665 lines to 10375 lines genotyped across a large set of markers. For all data sets, the use of the VBS-ML architecture improved genomic prediction accuracy over legacy linear based modelling approaches.

**Conclusions:**

An ML architecture governed under a variational Bayesian paradigm was shown to improve genomic prediction accuracy over legacy modelling approaches. This VBS-ML approach can be used to dramatically decrease the parameter burden on the network and provide a computationally feasible approach for improving genomic prediction conducted with large breeding population numbers and genetic markers.

## Introduction

Genomic Selection (GS), through genomic prediction, has proven to be a useful tool for achieving rapid genetic gain in livestock and plant breeding programmes. Since its inception in [1] genomic prediction approaches have mostly focused on using hierarchical linear models for assessing the relative genetic merit of lines for phenotypic traits of interest, with various prediction accuracies developed from these models [2]. Historically, these approaches were piecemeal, estimating proxy QTL effects using simple marker regression scans of the whole genome [1]. This was quickly extended to using the complete set of genetic markers in linear based models by considering the marker effects as random effects variables with various distributional properties [3–5]. When the number of markers became large, penalization methods such as the ones used in [3] and [4], became a useful tool for mechanistically pushing small marker random effects to zero and giving rise to various Bayesian variable selection methods [6, 7]. Once the number of markers became routinely larger than the number of individuals being studied, computationally efficient methods were developed that re-dimensionalised the genetic marker information in the models into an additive genomic relationship matrix (GRM) that allowed direct prediction of the relative performance of lines [8–12]. In more complex experimental scenarios, such as plant breeding programmes the inclusion of a dense GRM in a one-stage linear model can be computationally challenging due to requirements to involve the GRM in iterative parameter estimation algorithms [13, 14]. As the number of breeding lines and the number of testing environments increases the model becomes computationally cumbersome to solve [13] and modern computing techniques such as matrix algebra parallelisation [15–17] and machine learning (ML) approaches [18, 19] have become common place in GS research.

ML has now been widely adopted in crop and livestock agriculture when there is sufficient data complexity or computationally difficult tasks that require undertaking [20]. In the context of genomic prediction of agricultural traits, various deep learning techniques have been researched to understand their potential to improve prediction accuracy over legacy modelling approaches [18, 19]. These approaches use the complete set of genetic markers spanning the genome as input features to a neural network and the output, a trait of interest, is optimally learned through the network using various computationally intensive statistical modelling techniques. In most ML based genomic prediction cases the deep learning architecture has consisted of a type of artificial neural network, called a multi-layer perceptron (MLP), due to its ability to learn a high level of abstraction from the complex connection between the phenotype and genotype data [21–25]. In crops, such a maize and wheat, where grain yield or end use quality traits are highly quantitative in nature the use of MLP networks has also been shown to improve genomic prediction accuracy over more conventional ML approaches, such as convolutional neural networks [25–27].

Neural networks can be potentially complex and fixed architectural aspects of the network, such as the number of layers and the number of nodes (or neurons) within a layer, can be tuned in various ways to optimise the learning potential of the network [28]. In situations where the network becomes highly over-parameterised, various dropout techniques have been proposed for reducing the computational burden through the reduction of less important connections between layers [29, 30]. Historically, these techniques were based on random dropout of neurons or weights between layers [31, 32] but quickly expanded to more distributional based methods [33, 34] that include the use of regularizers such as the L1 or Lasso. Extensions of these regularizaton techniques are now focussing on using appropriate Bayesian hierarchical priors [35] to conduct variable selection of important markers in the initial stages of the network [36].

The objectives of this study were to evaluate the accuracy of a cutting edge ML based approach for conducting genomic prediction that involves the variational Bayesian sparsity (VBS) technique derived in [35] and L1-regularization for reducing the over-paramaterisation burden on the proposed MLP deep learning network. We have called this VBS-ML and to illustrate the effectiveness of the approach we conducted VBS-ML genomic prediction of grain yield collected from a large wheat breeding panel phenotyped for four years and genotyped with a high quality set of SNP markers. We compared the results of the newly proposed ML deep learning network with a more naive ML network as well as a more classical genomic prediction using linear mixed models (LMMs) and Bayesian regression methods BayesA and BayesB. In nearly all cases the VBS-ML network showed a marked improvement in genomic prediction accuracy compared to the naive ML network or other approaches. In addition, the genetic marker features selected from a given year or combined years were also shown to more accurately predict subsequent years compared to other prediction methods used in this research. This suggests the VBS-ML approach may potentially be a useful genomic selection tool for plant breeding programmes.

## Material and methods

### Plant material and phenotype data

Plant material used within this study consists of early and advanced generation breeding lines from within Australian Grain Technologies’ wheat breeding programmes. This material was spread across four field trials in the years 2014, 2016, 2017 and 2018. The 2014 field trial contained early and advanced breeding lines, comprised of material adapted for southern Australia (early and advanced lines) as well as material adapted for western and eastern Australia (advanced lines). A total of 10,375 genotypes were included in the trial and were planted in non-replicated randomised design with randomised grid checks (1 check per 11 plots). Further details of this trial can be found in [13]. Trials in 2016, 2017 and 2018 contained advanced breeding lines adapted to southern Australia. A total of 2869, 2869 and 2665 genotypes were included in the 2016, 2017 and 2018 trials respectively, with each of these trials planted in a completely randomised design with partial replication at a 1.25x level. All trials were sown as small-scale yield plots of 3 m^2^ at Roseworthy, South Australia ($-$34.52, 138.69), and managed according to best local practices. Phenotype data was collected as plot level grain yield from a mechanical small-scale plot harvester.

### Genotype data

In this research we used a whole genome set of genetic markers from a custom 20K SNP Affymetrix array that spanned the 21 chromosomes of the wheat genome. These markers are known to be of high quality and have been used as the basis for several published grains research articles [13, 14, 37]. To simplify the usage of the markers, we have used imputed markers only where missing alleles have been imputed using the *k*-NN nearest neighbor algorithm developed in [38] and used extensively in [39] [see 42, norm17]. For all methods below we define the genetic marker matrix for a set of lines in any given year as $\varvec{M} = [\varvec{m}_1\ldots \varvec{m}_p]$ of dimension *r* lines by *p* markers spanning the complete 21 wheat chromosomes.

### Adjusted yield derivation

Preceding genomic prediction using linear based models and ML we derive an adjusted yield prediction for each set of lines within a year using a spatial LMM that partitions and estimates genetic and non-genetic sources of variation. We specify this model in a general manner to allow for independent modelling of each trial conducted at Roseworthy. Let $\varvec{y}_e = [y_{e1}, \ldots , y_{en}]$ be the *n* raw yield observations from a field trial within a year. The LMM was then of the form

1 $$\begin{aligned} \varvec{y}_e = \varvec{1}_{n}\mu + \varvec{Z}_e\varvec{u} + \varvec{Z}_g\varvec{g} + \varvec{e}, \end{aligned}$$

where $\mu$ is the fixed grand mean parameter and $\varvec{1}_{n}$ is an *n* length vector of ones. The $\varvec{u}$ is a vector of random effects partitioned as $\varvec{u} = [\varvec{u}_1^T\ldots \varvec{u}_s^T]^T$ with conformably partitioned indicator matrix $\varvec{Z}_e = [\varvec{Z}_1\ldots \varvec{Z}_s]$. This partitioning is typically the result of including multiple random effect terms in the LMM that are required to account for non-genetic sources of variation, such as design induced effects or non-linear variation that may be occurring across the Rows or Range of the experiments. The complete set of random effects follow the distribution $\varvec{u} \sim N(\varvec{0}, \varvec{G})$ where $\varvec{G} = \oplus _{i = 1}^s\varvec{G}_i$ where $\oplus$ is the so-called direct sum structure that generates a block diagonal matrix and $\varvec{G}_i$ is typically simplified to $\sigma ^2_{ei}\varvec{I}_{n_i}$. Similarly the residual error term, $\varvec{e}$, was partitioned to $\varvec{e} = [\varvec{e}_1^T\ldots \varvec{e}_t^T]^T$ and distributed as $\varvec{e} \sim N(\varvec{0}, \varvec{R})$ where $\varvec{R} = \oplus _{i = 1}^t\varvec{R}_i$. Here, $\varvec{R}_i = \sigma ^2_i\varvec{\Sigma }^{(ro)}_i\otimes \varvec{\Sigma }^{(ra)}_i$ containing a paramaterization for a separable AR1 by AR1 (AR1 = autoregressive process of order 1) correlation process that adequately captures the similarity of the observations across distinct Range and Rows of the experimental design for the *it*th trial within a year. The final term on the right hand side of (1), contained a vector of genetic effects, $\varvec{g}$, of length *r* with an associated indicator matrix $\varvec{Z}_g$ that assigns the line to the appropriate yield plot in the experiment. The genetic effects capture the underlying genetic variation of yield across the breeding population around the experimental average $\mu$. The distribution of the effects are assumed to be $\varvec{g} \sim N(\varvec{0}, \sigma ^2_g\varvec{I}_r)$ where $\sigma ^2_g$ is the genetic variance.

Empirical best linear unbiased predictors (eBLUPs) of the genetic effects $\tilde{\varvec{g}}$ were then extracted from the fitted LMM and generalized heritabilities were calculated using [40]. The techniques of [41] were then used to conduct a de-regression to derive adjusted yield values for each line, namely

2 $$\begin{aligned} y_i = \hat{\mu } + \frac{\tilde{g}_i}{1 - PEV_i/\hat{\sigma }^2_g} , \qquad i = 1, \ldots , r. \end{aligned}$$

where $\tilde{\varvec{g}}_i$ and $PEV_i$ are the eBLUP and prediction error variance of the *i*th line respectively and $\hat{\sigma }^2_g$ is the Residual Maximum Likelihood (REML) estimate [see 42] of the genetic variance.

### Linear genomic prediction

We define a general form for the linear genomic prediction model of the adjusted yield, namely

3 $$\begin{aligned} \varvec{y} = \varvec{1}_n\mu ^* + \varvec{M}\varvec{q} + \varvec{e}^* \end{aligned}$$

where $\varvec{1}_n\mu ^*$ is the grand mean and $\varvec{q}$ is a *p* length vector of additive marker effects with a distribution that varies depending on the method used for prediction. In this reduced model the residual errors, $\varvec{e}^*$, are used to account for all non-additive genetic variation and have distribution $\varvec{e}^* \sim N(\varvec{0},\sigma ^2\varvec{I}_r)$.

#### LMM genomic prediction

For cases where the distribution of the marker effects are assumed to have an unconditional Gaussian of the form $\varvec{q} \sim N(\varvec{0}, \sigma ^2_a\varvec{I}_p)$, (3) can be defined as a genomic prediction LMM. If $p>> r$ then it is computationally convenient to re-write the LMM as

4 $$\begin{aligned} \varvec{y} = \varvec{1}_r\mu ^* + \varvec{a} + \varvec{e}^* \end{aligned}$$

where $\varvec{a}$ is an *r* length vector of additive line effects with distribution $\varvec{a} \sim N(\varvec{0}, \sigma ^2_a \varvec{G}_a)$. Here, $\sigma ^2_a$ is the additive genetic variance, $\varvec{G}_a= \varvec{M}\varvec{M}^T/s$ represents an $r \times r$ additive relationship matrix reflecting the marker based relationships between the lines and is scaled using $s=\text{ trace }(\varvec{M}\varvec{M}^T)/r$ [43].

Using the techniques of [44], (4) can be solved and the genomic predictions can be immediately written as

$$\begin{aligned} \tilde{\varvec{y}} = \varvec{1}_r\hat{\mu }^* + \tilde{\varvec{a}} \end{aligned}$$

where

$$\begin{aligned} \hat{\mu }^*&= (\varvec{1}^T_r\varvec{H}^{-1}\varvec{1})^{-1}\varvec{1}^T_r\varvec{H}^{-1}\varvec{y}\\ \tilde{\varvec{a}}&= \varvec{G}_a\varvec{H}^{-1}(\varvec{y} - \varvec{1}_r\hat{\mu }^*) \end{aligned}$$

where $\varvec{H} = \sigma ^2\varvec{I}_r + \sigma ^2_a\varvec{G}_a$. Typically, $\sigma ^2$ and $\sigma ^2_a$ are replaced by their Residual Maximum Likelihood (REML) estimates and $\tilde{\varvec{a}}$ becomes an empirically based additive genomic prediction of the adjusted yield.

#### BayesA and BayesB genomic prediction

BayesA and BayesB are a form of hierarchical Bayesian regression based on the linear model (3). In this model we now consider additional structure for the marker effects, $\varvec{q} = (q_1,\ldots , q_p)$ such that the *i*th marker effect has a distribution of the form

$$\begin{aligned} q_i\,|\,\sigma ^2_i, \pi&\sim \left\{ \begin{array}{ll} 0 &{} \text{ probability } \qquad \pi \\ N(0, \sigma ^2_i) &{} \text{ probability } \qquad 1 - \pi \end{array}\right. \\&\sigma ^2_i\,|\,\nu , s^2_q \sim \chi ^{-2}(\nu , s^2_q) \end{aligned}$$

where $\chi ^{-2}(\nu , s^2_q)$ represents a scaled inverse chi-square distribution with $\nu$ degrees of freedom and scale parameter $s^2_q$, or equivalently an $\Gamma ^{-1}(\nu /2, s^2_q\nu /2)$. After integrating over the marker variances, $\sigma ^2_i, i = 1, \dots , p$, we can obtain marginal marker effects of the form

$$\begin{aligned} q_i\,|\, \pi&\sim \left\{ \begin{array}{ll} 0 &{} \text{ probability } \quad \pi \\ t(0, s^2_q, \nu ) &{} \text{ probability } \quad 1 - \pi \end{array}\right. \qquad i = 1, \ldots , p. \end{aligned}$$

BayesB considers the complete structure derived here and BayesA is a special case of BayesB where $\pi = 0$. In both cases the non-zero marginal effects have a *t*-distribution with $\nu$ degrees of freedom and scale parameter $s^2_q$ reflecting the requirement to capture the important positive and negative marker effects and shrink negligible effects close to zero. The spike and slab prior of the marginal marker effects ensures BayesB acts like a feature selection method and consequently provides a useful comparison to the ML feature selection method outlined in the next section.

### ML genomic prediction

**Fig. 1:**
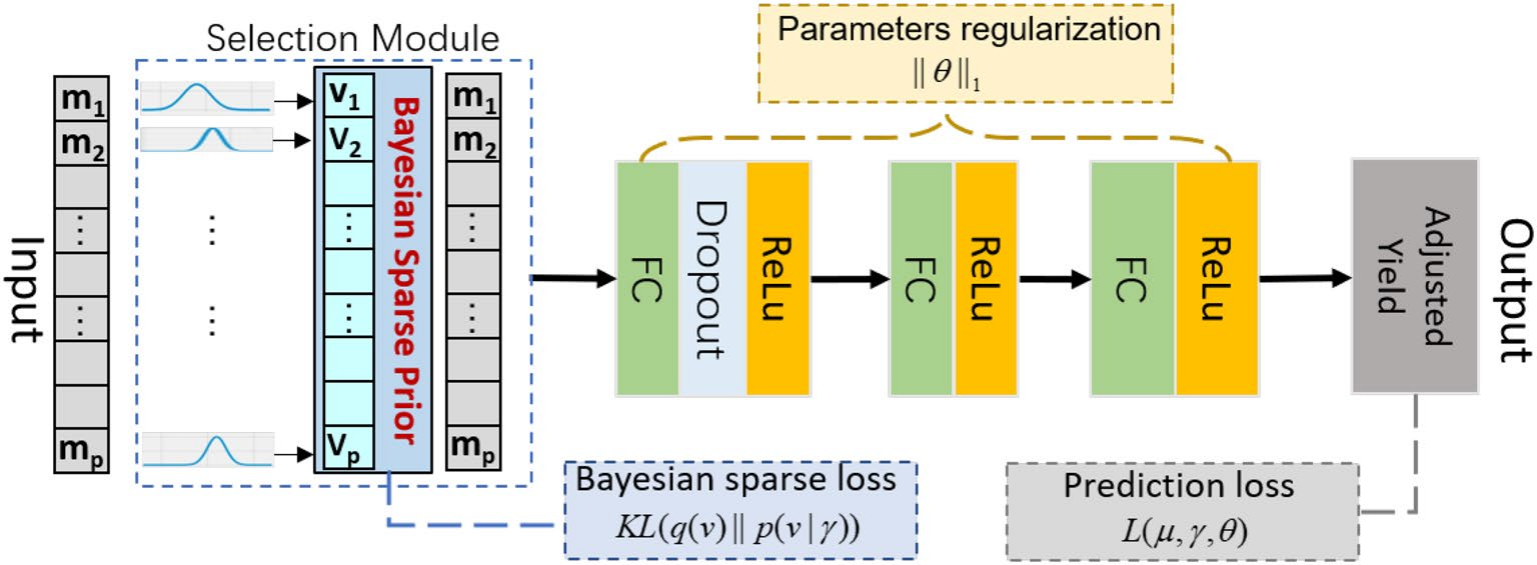
The proposed variational Bayesian ML architecture that includes the initial feature selection module and additional hidden layers in the prediction module

Based on its previous successful use in genomic prediction we have chosen to use an MLP-based machine learning scheme. The MLP is a densely connected network used in deep learning and is a typical feed-forward neural network that does not assume a particular structure of the input features [25]. We investigated the use of MLP architecture presented in Fig. 1. The MLP consisted of input layer that correspond to a fixed number of neurons where the complete set of neurons denote a set of SNP marker features from a row of $\varvec{M}$. The array of hidden layers then capture non-linear features from the output of the previous layers were each of the hidden layers may consist of varying number of neurons. Hidden Layers are usually fully connected (FC) between neurons with each connection given its own weight parameter. The output layer receives the outputs of the last hidden layer and provides the prediction, in this case a prediction of adjusted grain yield. The weights of the whole network are parameters that require learning from the training set and their estimates determine the effectiveness of each neurons contribution towards the final prediction. In the MLP architecture presented in Fig. 1, two major sub-networks are proposed including a feature selection module (for marker selection) and a prediction module (for result estimation). For each module, the component of the MLP model is described in more detail in the sections below along with a derivation of the objective function governing the complete network optimisation. For ease of notation we have considered a single sample in Fig. 1 with recognition this network is applied to all training samples.

#### Feature selection module

Let $\varvec{m} = [m_{1}, \ldots , m_{p}]^T$ represent a *p* length vector of genetic marker input features for a line or sample in the dataset. As shown in Fig. 1, we introduce a feature selection module to adaptively select the important genetic markers. Within this feature selection module we introduce a hidden selection layer with an output defined by the model

5 $$\begin{aligned} \varvec{x} = \varvec{m} \odot \varvec{v} \end{aligned}$$

where $\odot$ denotes the element wise multiplication and $\varvec{v} = [v_1, \ldots , v_p]^T$ is a vector of weights. The weights $\varvec{v}$ will be learned during network optimisation, and to enforce the sparsity of the selection, we assume they will be governed by a hierarchical sparse prior distribution. Before outlining the methodological details of this hierarchical prior and the associated learning objectives, we introduce the remainder of the neural network structures and operations.

#### Prediction module

After the selection module we then utilise further layers of an MLP (see Fig. 1) to refine the feature representation and prediction. Let $\varvec{w}_{j}$ be a *p* length vector of weights for the connections between the complete set of outputs from the first hidden layer to the *j*th neuron in the second hidden layer. The output for the *j*th neuron is then

6 $$\begin{aligned} z_{1j}= \text{ ReLU }\Bigl (b_{0}+ \sum _{s = 1}^p w_{js}x_{s}\Bigr ) \end{aligned}$$

where $b_0$ was the bias for the first hidden layer and $\text{ ReLU }$ denotes the the rectifier linear unit activation function. For a full set of connections between layers, (6) can be generalized to become

$$\begin{aligned} \varvec{z}_{1} = \text{ ReLU }(b_{0}+ \varvec{W}_{1}\varvec{x}) \end{aligned}$$

where $\varvec{x}$ is the *p* length vector of outputs from the first hidden layer and $\varvec{W}_{1}$ is a $(n_1 \times p)$ matrix with *j*th row $\varvec{w}_{j}$. Given an arbitrary *k* fully connected hidden layers the output from the *k*th hidden layer can be immediately written as

$$\begin{aligned} \varvec{z}_{k}= \text{ ReLU }(b_{k-1} + \varvec{W}_{k}\varvec{z}_{k-1}). \end{aligned}$$

where $b_{k - 1}$ and $\varvec{z}_{k - 1}$ is the bias and output from the $k - 1$ hidden layer and $\varvec{W}_{k}$ are the $(n_{k} \times n_{k - 1})$ matrix of weights. For the data sets used in this research we trained models using an MLP containing a prediction module with $k = 3$ FC hidden layers where $(n_1, n_2, n_3) = (256, 128, 1)$ with the last layer as the output layer. We utilized one dropout layer after the first layer.

#### Bayesian sparse prior for featue selection and objective function

Following [35], we assume the hierarchical sparse prior distribution for the feature selection weights $\varvec{v}$ is of the form

$$\begin{aligned} p(\varvec{v} \,|\, \varvec{\gamma })&= \prod _{i=1}^{p} p\left( v_{i} \,|\, \gamma _{i}\right) =\prod _{i=1}^{p} N\left( v_{i} \,|\, 0, \gamma _{i}\right) \\ p(\varvec{\gamma })&= \prod _{i=1}^{p} p\left( \gamma _{i}\right) =\prod _{i=1}^{p} \mathcal {U}\left( \gamma _{i} \,|\, a, b\right) . \end{aligned}$$

Here, $p(\varvec{v}\,|\, \varvec{\gamma })$ is the sparse prior for $\varvec{v}$ conditioned on the hyperparameters $\varvec{\gamma }$ and $\mathcal {U}\left( \gamma _{i} \,|\, a, b\right)$ is a uniform hyperprior with range hyperparameters [*a*, *b*]. The hyperparameters $\varvec{\gamma }$ will be estimated during the optimisation of the network.

For notational simplicity, we use $\mathcal {D}$ to indicate all the data samples (including both $\varvec{M}$ and $\varvec{y}$) available for training and let $\varvec{\theta }$ be the network parameters defined as the complete set of network weights with the exception of the feature selected weights, $\varvec{v}$. Under a Bayesian paradigm, we require the ability to learn the unknown variables or parameters $\varvec{v}$, $\varvec{\gamma }$, and $\varvec{\theta }$ from the given data $\mathcal {D}$ through an appropriate formulation of the posterior distribution $p(\varvec{v}, \varvec{\gamma }, \varvec{\theta } \,|\, \mathcal {D})$. Directly estimating this posterior is difficult. To make progress, we formulate the learning task with the variational Bayesian approach outlined in [33]. Firstly, we define a variational posterior for $\varvec{v}$ as $q(\varvec{v})= \prod _i q(v_{i})$ such that

7 $$\begin{aligned} q(v_{i}) = \mathcal {N}(\mu _{i}, \alpha _{i}\mu _{i}), \end{aligned}$$

where $\mu _{i}$ and $\alpha _{i}\mu _{i}$ are the mean and variance of the variational posterior, respectively. With (7), the proposed variational Bayesian learning task can be represented as

8 $$\begin{aligned} \min _{\varvec{\mu }, \varvec{\gamma }, \varvec{\alpha }, \varvec{\theta }} \text {KL}(q(\varvec{v}, \varvec{\gamma }, \varvec{\theta }) \,||\, p(\varvec{v}, \varvec{\gamma }, \varvec{\theta } \,|\, \mathcal {D}) ), \end{aligned}$$

where $\text {KL}$ denotes the Kullback–Leibler (KL) divergence, $\varvec{\mu }$ is used to parameterise the distribution corresponding to $\varvec{v}$, $p(\varvec{v}, \varvec{\gamma }, \varvec{\theta } \,|\, \mathcal {D})$ is the joint posterior distribution of the parameters given data, and $q(\varvec{v}, \varvec{\gamma }, \varvec{\theta })$ is the corresponding variational joint posterior of $p(\varvec{v}, \varvec{\gamma }, \varvec{\theta } \,|\, \mathcal {D})$.

We assume $\varvec{\theta }$ and $\varvec{v}$ in the prior distribution are independent and this allows the joint posterior to be re-formulated as

$$\begin{aligned} p(\varvec{v}, \varvec{\gamma }, \varvec{\theta }) = p(\varvec{v}, \varvec{\gamma })p(\varvec{\theta }). \end{aligned}$$

By using the variational posterior to approximate the true posterior, the objective in (8) can be re-formulated as the variational lower bound (VLB) of the marginal likelihood [45] over the data, namely

$$\begin{aligned} \min _{\varvec{\mu }, \varvec{\gamma }, \varvec{\alpha }, \varvec{\theta }} \mathcal {L}(\varvec{\mu }, \varvec{\gamma }, \varvec{\theta }) - \text {KL}(q(\varvec{v}) \,||\, p(\varvec{v}\,|\,\varvec{\gamma }) ) - \text {KL}(q(\varvec{\gamma }) \,||\, p(\varvec{\gamma })) - \text {KL}(q(\varvec{\theta }) \,||\, p(\varvec{\theta })), \end{aligned}$$

where $\mathcal {L}$ denotes the expected log-likelihood and absorbs the loss term for optimally fitting the data. [35] shows that under a uniform hyperprior for $\varvec{\gamma }$, $\text {KL}(q(\varvec{\gamma }) \,||\, p(\varvec{\gamma }))$ does not depend on $\varvec{\mu }, \varvec{\gamma }, \varvec{\alpha }$ or $\varvec{\theta }$ and can be safely ignored. Assuming $p(\varvec{\theta })$ is a known Laplacian distribution of the form $Laplacian(\varvec{\theta }\,|\,0, \lambda _\theta )$ with hyperparameters $\lambda _\theta$, we can now reduce the VLB to

$$\begin{aligned} \min _{\varvec{\mu }, \varvec{\gamma }, \varvec{\alpha }, \varvec{\theta }} \mathcal {L}(\varvec{\mu }, \varvec{\gamma }, \varvec{\theta }) -\lambda _{\theta } \Vert \varvec{\theta }\Vert _1 - \text {KL}(q(\varvec{v}) \,||\, p(\varvec{v}\,|\,\varvec{\gamma })), \end{aligned}$$

where $\lambda _{\theta } \Vert \varvec{\theta }\Vert _1$ is the derived regularizer of $\varvec{\theta }$. Using the results from [35] as well as a mean absolute error loss function we can then derive a final objective function for jointly estimating feature selection weights $\varvec{v}$ and the network parameters $\varvec{\theta }$, namely

9 $$\begin{aligned} \mathcal {L}_\text {obj} = \sum _{i=1}^{r}|y_i- \tilde{y}_i|/ n + \lambda _{\theta } \Vert \varvec{\theta }\Vert _1 + 0.5\sum _{i=1}^p \text {log}(1+\alpha _{i}^{-1}), \end{aligned}$$

where $y_i$ is the adjusted yield and $\tilde{y}_i$ is the predicted yield for the *i*th sample. The final term on the right hand side of (9) can be viewed as the variational Bayesian sparsity regularization term to encourage sparsity of the feature selection weights across the *p* dimensions. The estimated means for the posterior of $\varvec{v}$, $\varvec{\mu }$, will be used as the actual sparse weights for feature selection and the parameters $\alpha _{i},\, i = 1, \ldots , p$ control the sparsity of these weights. This derived component then acts as a regularizer for the weights $\varvec{v}$ where, for example, if $\alpha ^{-1}_i \rightarrow 0$ during training, then the corresponding weight $v_{i}$ and the associated feature/marker $m_i$ from any given $\varvec{m}$ can potentially be ignored in the subsequent processes of the neural network. After optimisation, a set of sparse weights are have been automatically and adaptively learned. For this reason the feature selection does not need a manually set threshold. This complete ML approach we have called VBS-ML.

### Computations and Benchmarking

The LMM was fitted using the flexible LMM R package ASReml-R [46] available in the R statistical computing environment [47] and commercially available from VSNi at https://vsni.co.uk/software/asreml-r. For computational efficiency we incorporated the genetic marker relationship matrix of the lines through the special function vm() in the random model formula.

BayesA and BayesB models were computationally fitted using the BGLR R package [48] freely available in the R statistical computing environment [47]. Due to the intractability of the posterior density of the parameters for both hierarchical models, BGLR uses a numerically based Gibbs sampling algorithm. BGLR also assumed some additional structure of some hyperparameters that included $\pi \sim Beta(\pi _0, p_0)$ where we have assumed the probability of marker inclusion to be $1 - \pi _0 = 0.05$ and $p_0$ is sufficiently large to ensure $E(\pi ) = \pi _0$. Additionally, we have assumed $\nu = 4$ and $s^2_q$ is assumed to be distributed $s^2_q \sim \Gamma (s, r)$ where $s = 1.1$ and solved for the rate parameter based on an attributed $R^2 = 50\%$ (R-squared) for the linear predictor $\varvec{M}\tilde{\varvec{q}}$. Other MCMC numerical attributes such as number of total iterations, burn in number of iterations and thinning were set at default values.

For the ML networks we used the Pytorch [49] package available in the Python software environment [50] where we assumed a batch size of 512 and $1e^5$ epoch. We used an ADAM optimiser and a cosine annealing learning rate adjustment strategy with a learning rate of $1e^{-4}$ and a weight decay of $5e^{-4}$. We set $\lambda _\theta$ as $1e^{-3}$. For the ADAM optimisation we used $\beta _1$=0.9, $\beta _2$=0.99. Our network had four fully connected layers and three residual blocks.

For computational benchmarking, we focussed on computational timings for conducting analyses of the 2014 and 2016 data sets only. The 2017 and 2018 data sets are very similar in size to 2016 and would generate redundant information. For the linear genomic prediction approaches we used an Oracle cloud instance (OCI) with 16 OCPU and 256 Gb RAM. For the ML networks, we used an OCI consisting of 12 OCPU with 72Gb RAM and a NVIDIA Tesla P100 with 3584 cores.

### Model validation and accuracy

We randomly partitioned the complete data set into training and validation data sets four times. For each split we used a training data set containing 90% of the samples and a validation data set with the remaining 10% of the samples. Training and testing data sets did not overlap. For each split, the models were trained on the training data set only and the accuracy of the genomic prediction was assessed on the validation set.

There has been some recent discussion on the sole use of Pearsons correlation for asessing accuracy [51, 52] when regularization or feature selection methods are used for genomic prediction. For this reason, we have used a combination of Pearsons correlation and relative accuracy (RE) where, for *n* samples, the RE was defined as

$$\begin{aligned} RE = \frac{1}{n}\sum _{i=1}^n |y_i - \tilde{y}_i|/y_i \end{aligned}$$

The use of the observed value in the denominator of each of the elements provides a mechanism to scale the error according to the size of the observations that are being predicted. This RE provides an easily intepretable average proportional difference between the predicted and observed values.

**Fig. 2:**
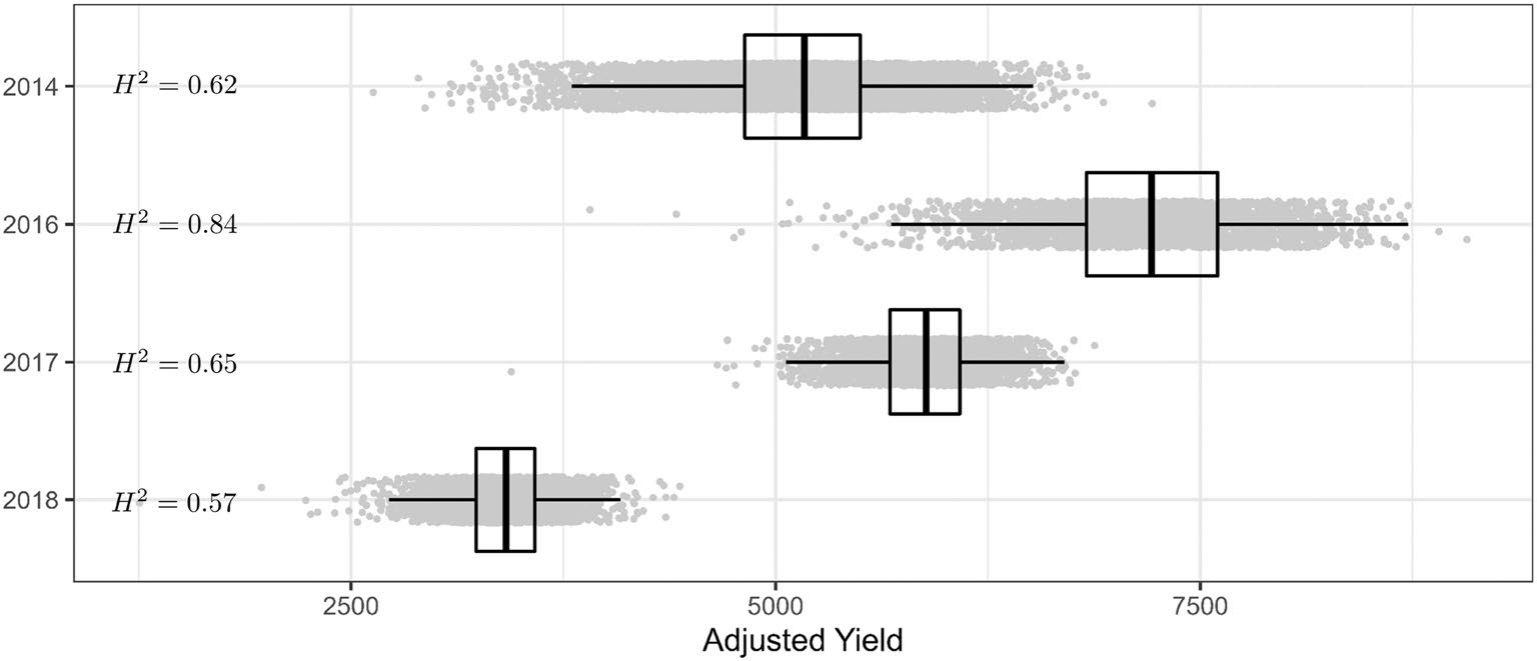
Distribution of the derived adjusted yield across the set of lines for Roseworthy data sets from 2014, 2016, 2017 and 2018. Generalized broad sense heritabilities $H^2$ are given on the left hand side of the plot

## Results

Figure 2 presents the distribution of the adjusted yield values for each of the years. The plot indicates the large differences in average yield across the lines over the years used in this research even though they were similarly located. The variation of the adjusted yield values for 2014 and 2016 were similar with reduced variation in 2017 and 2018. The broad sense heritabilities for each of the years indicate yield is under strong genetic influence across the set of varieties in each year. This suggests there are definitive underlying mechanisms for the changes in yield between varieties and these can be modelled using genomic prediction.

**Fig. 3:**
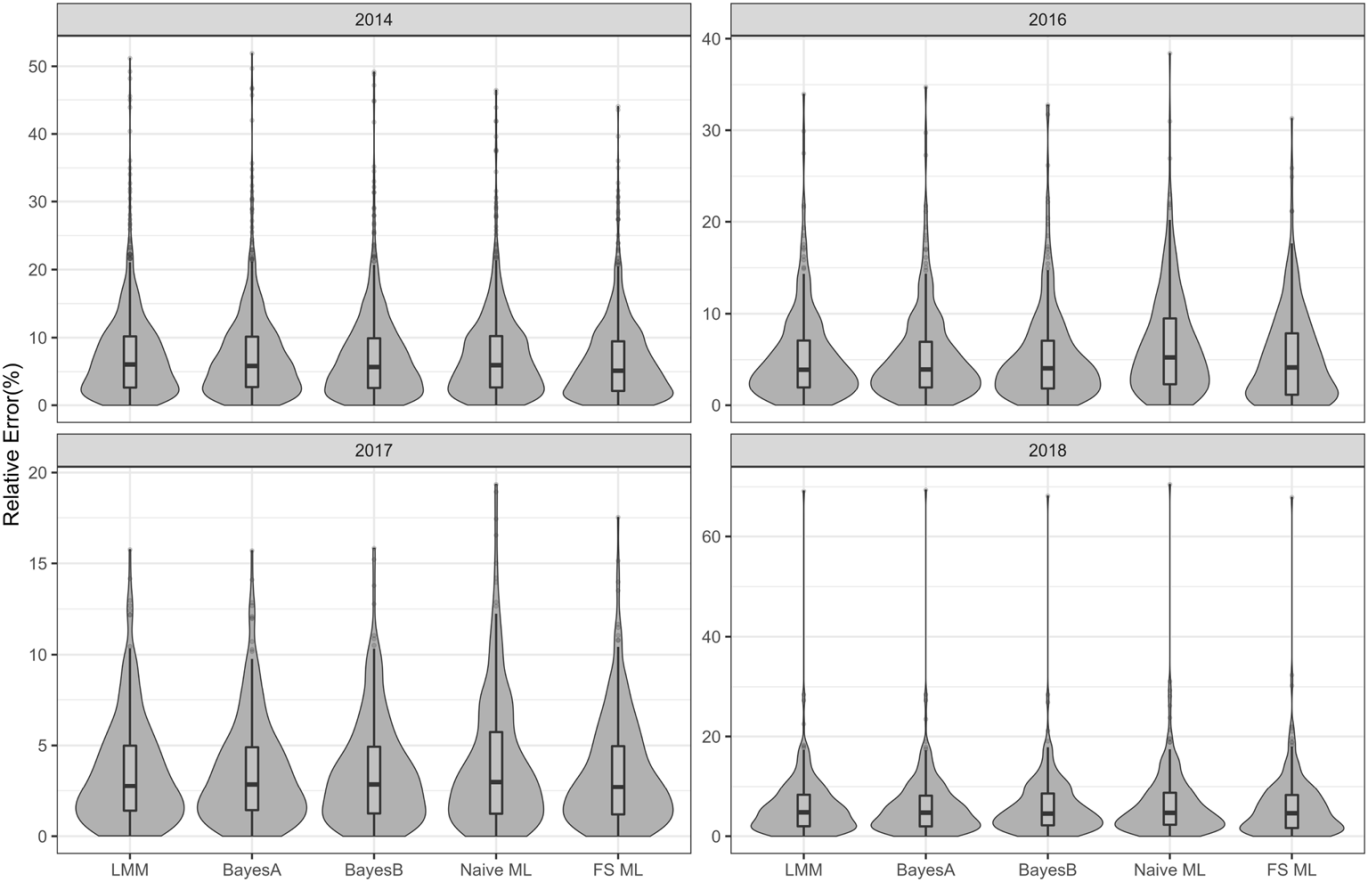
Relative error prediction accuracy for genomic prediction methods LMM, BayesA, BayesB, Naive-ML and VBS-ML conducted on split 1 of the Roseworthy data sets from 2014, 2016, 2017 and 2018

**Table 1:** For each of the data sets, the mean relative errors (%) from each of the genomic prediction methods conducted using four random cross-validation splits with 90% training data and 10% validation data. The number of feature selected markers for VBS-ML is given in parentheses

Year	Methods	1	2	3	4	Ave.
2014	LMM	7.38	7.21	7.44	7.31	7.34
	BayesA	7.28	7.16	7.37	7.25	7.27
	BayesB	7.20	7.09	7.36	7.21	7.22
	Naive-ML	7.61	7.56	8.31	7.61	7.77
	VBS-ML (354)	6.49	7.08	7.58	7.04	7.05
2016	LMM	5.36	5.00	5.11	5.23	5.18
	BayesA	5.36	5.02	5.15	5.20	5.18
	BayesB	5.30	5.02	5.07	5.28	5.16
	Naive-ML	6.67	6.28	6.59	6.10	6.41
	VBS-ML (409)	5.20	4.89	4.94	5.04	5.02
2017	LMM	3.53	3.37	3.63	3.45	3.50
	BayesA	3.54	3.38	3.64	3.48	3.51
	BayesB	3.51	3.43	3.65	3.43	3.51
	Naive-ML	3.99	4.16	4.18	3.77	4.03
	VBS-ML (315)	3.48	3.26	3.54	3.26	3.39
2018	LMM	5.94	4.98	5.80	5.94	5.67
	BayesA	5.94	4.99	5.77	5.94	5.66
	BayesB	5.98	5.06	5.63	5.97	5.66
	Naive-ML	6.39	5.70	6.20	6.23	6.13
	VBS-ML (385)	5.89	4.98	5.16	5.86	5.47

### Linear genomic prediction approaches achieve similar accuracy

For each of the data sets, Table 1 presents the mean relative errors from each of the genomic prediction methods conducted using four random cross-validation splits with 90% training data and 10% validation data. Additionally, to visually gauge the accuracy variation, Fig. 3 presents the relative error across the complete set of lines for each genomic prediction method by year combination for split 1 only. Table 1 and Fig. 3 indicate, across most splits and years, the linear regression approaches LMM, BayesA and BayesB produced very similar results with BayesA and BayesB slightly outperforming LMM. Notably, for the 2016, 2017 and 2018 data sets, Table 1 indicates that the Bayesian regression approaches only produced negligible improvements or no improvement at all over the legacy LMM approach potentially indicating that using a smaller number of lines may impact the ability for these hierarchical models to improve the accuracy of the prediction.

### VBS-ML improves relative accuracy over all other approaches

Table 1 and Fig. 3 definitively show that the VBS-ML approach achieves the lowest relative error compared to all other approaches used here. This reduction occurred even though the number of markers used in the prediction component of the VBS-ML network was reduced through feature selection by up to 98%. These relative error reductions are close to 0.2% for VBS-ML compared to LMM, BayesA, BayesB and between 0.6% and 1.4% for VBS-ML compared to the Naive-ML approach. Additionally, Fig. 3 indicates the VBS-ML tends to have a higher relative error peak closer to zero with thinner tails generated from the larger relative errors. Table 1 also indicates that, on average, for all splits and years, the Naive-ML approach was definitively the poorest performing genomic prediction approach compared to all others. In many cases the relative error increase using the Naive-ML were $> 1\%$ for some splits and this equates to a considerable difference on the scale of the response. For example, from split 3 in 2014 a relative error increase of 0.87% using Naive-ML compared to LMM equates to a 44 kg/ha increase in the average differences between the predicted and observed yield values. Figure 3 also indicates that, compared to other methods, the distribution of relative errors for Naive-ML tends to have a smaller peak further away from zero and a fatter tail. This skewness is especially prevalent for the relative errors in 2016 where there were dramatic differences between Naive-ML and other approaches.

**Fig. 4:**
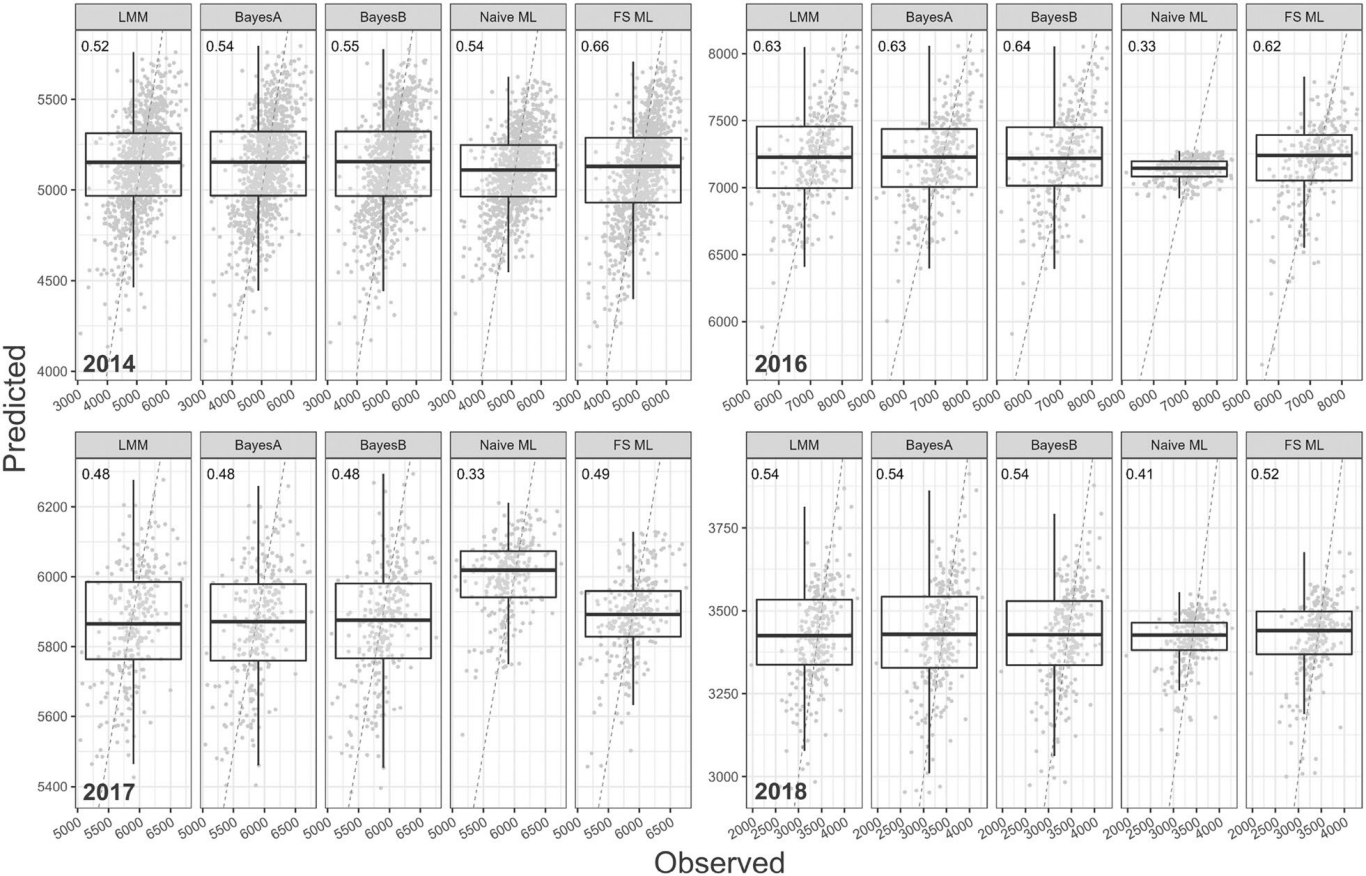
Relative error prediction accuracy for genomic prediction methods LMM, BayesA, BayesB, Naive-ML and VBS-ML conducted on split 1 of the Roseworthy data sets from 2014, 2016, 2017 and 2018

**Table 2:** For each of the data sets, the Pearson correlation between the observed and predicted grain yield from each of the genomic prediction methods conducted using four random cross-validation splits with 90% training data and 10% validation data. The number of feature selected markers for VBS-ML is given in parentheses

Year	Methods	1	2	3	4	Ave
2014	LMM	0.52	0.49	0.50	0.47	0.50
	BayesA	0.54	0.50	0.51	0.48	0.51
	BayesB	0.55	0.51	0.51	0.49	0.51
	Naive-ML	0.54	0.41	0.39	0.42	0.44
	VBS-ML (354)	0.66	0.50	0.47	0.52	0.54
2016	LMM	0.63	0.56	0.65	0.57	0.60
	BayesA	0.63	0.56	0.65	0.58	0.61
	BayesB	0.64	0.55	0.65	0.57	0.60
	Naive-ML	0.33	0.24	0.41	0.33	0.33
	VBS-ML (409)	0.62	0.53	0.68	0.56	0.60
2017	LMM	0.48	0.52	0.53	0.52	0.51
	BayesA	0.48	0.52	0.51	0.51	0.51
	BayesB	0.48	0.51	0.51	0.53	0.51
	Naive -ML	0.33	0.38	0.40	0.52	0.41
	VBS-ML (315)	0.49	0.55	0.54	0.60	0.54
2018	LMM	0.54	0.54	0.46	0.48	0.51
	BayesA	0.54	0.54	0.47	0.47	0.50
	BayesB	0.54	0.54	0.49	0.46	0.51
	Naive-ML	0.41	0.25	0.32	0.37	0.34
	VBS-ML (385)	0.52	0.50	0.57	0.44	0.51

### VBS-ML slightly improves correlation

Table 2 presents the Pearsons correlations of the predicted vs the observed values of grain yield for each of the genomic prediction methods conducted on each data set from four random cross validation data splits. To complement the table, Fig. 4 presents the correlation of the predicted vs observed grain yield values obtained from all genomic prediction methods conducted using split 1 of each data set. Table 2 indicates that, on average, VBS-ML generated similar correlation to the linear regression methods for the 2016 and 2018 data sets. For the 2014 and 2017 data sets VBS-ML managed to slightly improve over these approaches. This is especially evident in the 2014 correlation plot in Fig. 4 where there appears to be a broader and stronger relationship. The table also indicates, across all data sets, the linear regression approaches achieved a very similar correlation. This similarity is also highlighted in Fig. 4 where the median values and distribution of the predicted values is similar from all three prediction methods. Further to the discussion of relative error, Table 2 and Fig. 4 indicate the Naive-ML genomic prediction method for each year had substantially reduced correlations in 2016, 2017 and 2018 and for 2017 it also induced a mean shift in the predicted values.

**Table 3:** Mean relative error prediction accuracy (%) for genomic prediction of an MLP that used feature selected markers from one year to predict adjusted grain yield in future years

Approach	1	2	3	4	Ave.
2016 $\Longrightarrow$ 2017	3.49	3.27	3.55	3.27	3.40
2016 $\Longrightarrow$ 2018	5.90	5.13	5.17	5.84	5.51
2017 $\Longrightarrow$ 2018	6.32	5.83	5.82	6.02	6.00

### Feature selected markers show useful predictive properties

The connectivity of the breeding lines between years 2016 and 2018 allows us to further verify the effectiveness of the proposed selection module for genomic prediction. After conducting ML genomic prediction independently in 2016 and 2017, we used the feature selected markers from each of the years to train an MLP to predict adjusted grain yield in future years. Table 3 shows the relative error genomic prediction accuracy of an MLP where feature selected markers from 2016 are used to predict adjusted grain yield in 2017 and 2018 and where feature selected markers from 2017 are used to predict adjusted grain yield in 2018. Comparing this table to the relative error prediction accuracies in Table 1 indicates that using an MLP consisting of feature selected markers from 2016 to predict 2017 adjusted grain yield managed to outperform all genomic prediction methods, except for VBS-ML, conducted on 2017 data. A similar result was observed from the prediction of 2018 adjusted grain yield from feature selected markers in 2016 with improved accuracy from VBS-ML when using only 2018 data. When an MLP, consisting of 2017 feature selected markers, was used to predict 2018 adjusted grain yield data, the relative error slightly improved over the Naive ML approach using 2018 data but was outperformed by all other genomic prediction methods used with the 2018 data.

**Fig. 5:**
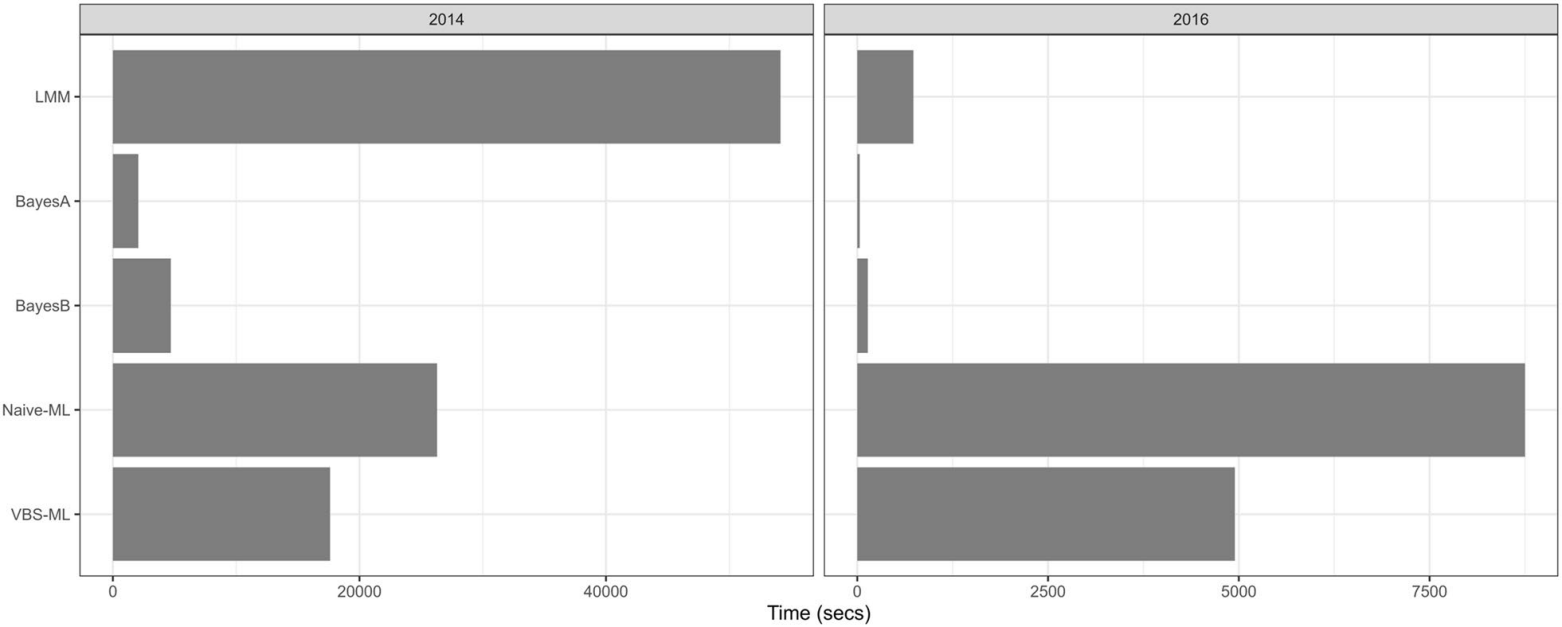
Computational timings of each of the analysis methods for split 1 of the 2014 and 2016 data. Timings are in seconds of elapsed CPU or GPU time

### VBS-ML shows efficiency over LMM for large data sets

Figure 5 presents the computational timings for the analysis methods conducted in the OCIs. The inflated computational time of the LMM in 2014 is due to the ASReml-R 4.1 version used in this research that can only conduct the LMM optimisation on one core of the 16 available in OCI. The large reduction in LMM computational time for 2016 is due to the large reduction in size of the relationship matrix being used in the optimisation. BayesA and BayesB are clearly the most computationally efficient analysis methods for both data sets. Both approaches utilised all 16 cores available in the OCI suggesting that the MCMC approach implemented in the software is highly parallelized.

For the ML networks, although an OCI consisting of 12 OCPU with 72Gb RAM was available for use in tandem with the NVIDIA Tesla P100, only one CPU with 20% of the available CPU RAM was needed to analyse the 2014 data. For the smaller data set in 2016 the linear prediction analysis approaches computationally outpaced the VBS-ML and Naive-ML analysis methods. For the much larger data set in 2014, the Naive-ML is 2$\times$ faster than the LMM approach and the VBS-ML is 3$\times$ more efficient. Although highly parallelized through the Tesla multi-core GPU, the ML approaches were not as efficient as multi-core CPU BayesA and BayesB.

## Discussion

This research focussed on improving prediction accuracy in large scale genomic prediction problems using an MLP architecture consisting of a feature selection module governed by variational Bayesian sparsity inference. For all data sets analysed in this study the number of genetic markers exceeded the number of samples. Consequently, the incorporation of the feature selection module in the initial stages of the ML architecture provided clear benefits through dramatically reducing the number of important markers and the burden of over-paramaterisation on the network. Further reductions in the over-parameterisation were achieved through the use of an $L_1$ penalty on the weights of the network across the hidden layers. The VBS-ML approach was shown to improve genomic prediction accuracy over linear based legacy genomic prediction approaches such as LMM, BayesA and BayesB as well as the naive MLP without the feature selection module. In addition, we showed the feature selection of markers obtained from one year could be used to train an MLP for the following years data and produce a competitive accuracy that would usually outperform legacy based approaches trained on the year that was being predicted.

The VBS-ML analysis approach can be considered to be an embedded feature selection approach that ensures redundant SNP markers are removed and the markers with the highest association in each linkage disequilibrium grouping are retained [53, 54]. This suggests this approach would be broadly applicable to other traits beyond grain yield where polygenicity or genetic complexity varies. Additionally, the feature selection properties of the VBS-ML can be considered to provide *explainability* of the prediction through identification of important contributing markers [55].

The VBS inference governing the initial layer of the MLP architecture is akin to the application of variable selection in more traditional regression problems where the objective is optimisation of a non-concave penalized likelihood [56]. Specifically, the resulting log penalty that is derived from the hierarchical modelling of the feature selection weights resembles log penalties derived in various variable selection studies [57, 58]. This penalty is well known for generating sparse solutions when it is applied to coefficients associated with a large set of covariates and a similar result was observed with the feature selection weights associated with the VBS-ML method used in this research. Penalties of this type have the so-called oracle property described by [56] that ensures strong sparsity without loss of accuracy for non-zero weights or coefficients over more traditional estimation approaches. In this case the penalty is inferred by the distributional hierarchy of the weights but this also suggests other non-hierarchical oracle type penalties, such as the extended penalty class in [58] could be used in the initial layers of the MLP. This is now being explored and is a subject of further research.

In this study we focussed on improving the additive component of genomic prediction using ML. We note that [36] used a comparative Bayesian variable selection type ML architecture that attempted to incorporate epistatic features but had limited success in improving genomic prediction over more standard approaches. [59] used strong regularization of a small number of ML network weights in an approximate Bayesian setting to ensure over-parameterisation of the network was reduced and slightly improved genomic prediction accuracy through estimation of additive components of epistasis. We are now exploring the use of a novel VBS-ML approach to efficiently incorporate and select important non-additive features that will include epistatic as well higher order features that would not usually be modelled through legacy approaches.

## Conclusion

The novel VBS-ML method discussed in this research provides a computationally feasible approach for undertaking genomic prediction modelling when the data contains large numbers of lines phenotyped and genotyped across a large set of genetic markers. This approach is of particular relevance to the plant breeding community where there has been a sizeable increase in the germplasm sets being used for genomic analyses [13, 14] and current analysis software limitations are being reached. The high parallelisation of the ML predictive task will require plant breeding organizations to acquire appropriate computational infrastructure as well as analytically integrate the VBS-ML into their plant breeding pipelines. If this is achieved, this research indicates that VBS-ML may be a useful avenue for improved genomic prediction accuracy, allowing plant breeders to accelerate their breeding cycles and continue to increase rates of genetic gain.

## Data Availability

The phenotype and genotype datasets for the 2014 Roseworthy trial are publicly available from the Supplmentary material of the article https://link.springer.com/article/10.1007/s00122-017-2975-4 and also available for direct download from https://doi.org/10.25909/23949333.v1. The phenotype and genotype datasets for the 2016, 2017 and 2018 Roseworthy trials are under commercial IP arrangements and not publicly available at this point. The python VBS-ML implementation is publicly available and downloadable from the GitHub repository, https://github.com/mfruzan/VBS-ML-Genomic-Prediction. In addition the R scripts to conduct the ASreml-R and BGLR analyses are also available along with information about the 2014 split identification data that was used to generate the results in this manuscript.
